# Telling Friend from Foe: Listeners Are Unable to Identify In-Group and Out-Group Members from Heard Laughter

**DOI:** 10.3389/fpsyg.2017.02006

**Published:** 2017-11-16

**Authors:** Marie Ritter, Disa A. Sauter

**Affiliations:** Department of Social Psychology, University of Amsterdam, Amsterdam, Netherlands

**Keywords:** laughter, groups, emotion, in-group advantage, motivation

## Abstract

Group membership is important for how we perceive others, but although perceivers can accurately infer group membership from facial expressions and spoken language, it is not clear whether listeners can identify in- and out-group members from non-verbal vocalizations. In the current study, we examined perceivers' ability to identify group membership from non-verbal vocalizations of laughter, testing the following predictions: (1) listeners can distinguish between laughter from different nationalities and (2) between laughter from their in-group, a close out-group, and a distant out-group, and (3) greater exposure to laughter from members of other cultural groups is associated with better performance. Listeners (*n* = 814) took part in an online forced-choice classification task in which they were asked to judge the origin of 24 laughter segments. The responses were analyzed using frequentist and Bayesian statistical analyses. Both kinds of analyses showed that listeners were unable to accurately identify group identity from laughter. Furthermore, exposure did not affect performance. These results provide a strong and clear demonstration that group identity cannot be inferred from laughter.

## Introduction

Group membership is important for how we perceive others: Across a range of domains, people perform better when processing information from in-group members. For example, we attend more closely to faces from our own group (Byatt and Rhodes, 2004), we are better at recognizing the identity of in-group members (Hehman et al., 2010), and we are more accurate in identifying emotions from non-verbal expressions produced by members of our own group (Elfenbein and Ambady, 2002). In some cases, the *belief* that another is a member of the perceiver's own group is sufficient to confer these advantages. In a study by Thibault et al. (2006), participants were asked to identify the emotion on faces that participants were told belonged either to their own or to another, group. When participants thought that they were making judgments about an in-group member, they were better at recognizing the expressed emotion, regardless of the actual group membership of the expresser. This lends support to the motivational account, which explains the performance advantage for in-group members as the result of greater motivation to process information from in-group members more deeply (Thibault et al., 2006). If we think that someone is a member of our own group, we are thus more motivated to, for example, find out what they are feeling. In order for this motivational mechanism to operate, the perceiver first has to be able to accurately judge whether the other person is a member of their own group. In the current study, we aimed to test whether listeners can discern group membership from hearing non-verbal expressions, specifically laughter.

Most research to date that has examined group membership has studied visual, rather than auditory perception. Visual experimental stimuli often contain clear features that distinguish groups, such as skin color (Cassidy et al., 2011). However, even for visual perception, determining group membership is not always entirely straightforward. In one study, Marsh et al. (2003) presented American participants with pictures of American–Japanese (American citizens with Japanese heritage) and Japanese (Japanese citizens with Japanese heritage) people, who posed with either neutral or emotional expressions. Participants were asked to categorize the pictures according to whether they thought the person was American–Japanese or Japanese. Participants performed better when judging the emotional expressions, as compared to the neutral expressions, suggesting that the emotional expressions may contain information about group membership akin to an accent in speech (e.g., Clopper and Pisoni, 2004b). Indeed, studies suggest that while observers agree on prototypical expressions of specific emotions (e.g., Ekman and Friesen, 1978), they also show culture-specific differences in how they express emotions, which has been dubbed emotion dialects (Elfenbein et al., 2007). These emotion dialects may be what perceivers use to infer group membership (Marsh et al., 2003), which then affects emotion recognition accuracy. However, less work has examined group membership inferences from vocal expressions beyond language.

For language-like vocal expressions, even brief vocal segments can convey group membership, as shown by Walton and Orlikoff (1994). They found that people could identify the ethnicity of a speaker 60% of the time from *a* sounds alone. More recently, Bryant et al. (2016) found that listeners could infer information about social relationships from human laughter. Specifically, listeners could identify whether people laughing together were friends or strangers. This suggests that human non-verbal vocalizations convey some information about social relationships, and perhaps might also carry group information. This would also be in line with research on chimpanzee calls, which has found that chimpanzees adjust their calls to distinguish themselves from close living groups (Crockford et al., 2004), and that these differences are meaningful to listeners (Herbinger et al., 2009).

Laughter is arguably the most extensively researched human non-verbal vocalization (Owren and Amoss, 2014). It occurs frequently, typically in social situations (Provine, 2004; Scott et al., 2014). Although different forms of laughter can communicate a range of social messages (Szameitat et al., 2010; Wildgruber et al., 2013), laughter is recognized across cultures as indicating amusement (Sauter et al., 2010). There are many different types of laughter, such as joyous, taunting, or tickling laughter, that seem to play distinct roles in social cognition (Szameitat et al., 2010; Wildgruber et al., 2013). Laughter can function as a signal of affiliation (Bryant et al., 2016), and may even constitute an extended form of grooming, through which social bonds are maintained and strengthened (Dezecache and Dunbar, 2004). Laughter thus presents a good candidate for examining group membership identification, given its ubiquity, sociality, and occurrence across cultures.

Only a single study to date has examined whether listeners can infer group membership from human non-verbal vocalizations. Sauter (2013, Experiment 1) tested Dutch participants' perception of vocalizations expressing amusement, relief, triumph, and sensual pleasure. The stimuli were from three different countries: the Netherlands (in-group), England (close out-group), and Namibia (distant out-group). Participants were first asked to classify the expressed emotion, and then to identify whether the person was from the Netherlands, another European country, or a country outside Europe. In the emotion recognition task, an in-group advantage was found, meaning that participants were more accurate in judging emotional expressions from members of their own cultural group. In contrast, participants were no better than chance at identifying group membership.

This result casts doubt on whether non-verbal vocalizations of emotion provide reliable group membership information. However, it is worth noting some limitations of Sauter's (2013) study: Firstly, it included vocalizations of multiple emotions. While this was necessary to test the in-group advantage for emotion recognition, it may have increased task difficulty in the group classification task. Secondly, the study by Sauter only included one nationality per group. This could have resulted in participants performing poorly due to the fact that they were unable to, for example, distinguish in-group from close out-group, even though they may have been able to accurately differentiate, for example, in-group from distant out-group. Thirdly, the study by Sauter employed only frequentist statistical analyses, which cannot provide support for a null hypothesis. The current study sought to remedy those limitations in order to provide a tougher test of the question of whether listeners can judge group membership from non-verbal vocalizations of emotion. We further sought to examine a potential role for familiarity in group identification judgments.

Although there is little evidence on the impact of familiarity on group identification in the context of non-verbal emotional expressions, studies of language perception point to a link between familiarity and accuracy for group identification (see Elfenbein and Ambady, 2002 for a similar result for emotion recognition). In one study, participants who had lived in many different US states were better at telling from which state a speaker came, compared to participants who had lived in one state for most of their lives (Clopper and Pisoni, 2004a). Baker et al. (2009) found a similar pattern in a study of the perceptions of an accent from the American state Utah. They found that participants who were from a state close to Utah (i.e., a close out-group), were nearly as good as the Utahans (i.e., members of the in-group), at identifying a Utahan accent. In contrast, participants from more distant states (i.e., the distant out-group), performed considerably worse, which was explained as being due to low familiarity with the Utahan accent. These results point to familiarity as a possible factor in group identification from vocal cues, and we therefore included a measure of exposure to other cultures in the current study, in order to test this possibility directly.

### The current study

The current study sought to examine whether listeners could identify in- and out-group members from laughter segments. Following Sauter (2013), we employed nationality as a proxy for group membership, as national identity is a salient and reliable group dimension (Smith, 1991). In addition, we distinguished between in-group, close out-group, and distant out-group (Sauter, 2013).

In examining the question of whether listeners would be able to identify group membership from laughter, we made the following predictions, based on the literature reviewed above: We hypothesized that listeners would be able to distinguish between laughter from different nationalities (Specific Group Identification Hypothesis). We further predicted that listeners would be able to accurately judge whether a laughing person belonged to the listener's own in-group, a close out-group, or a distant out-group (Broad Group Identification Hypothesis). Finally, we predicted that greater exposure to laughter from members of other cultural groups would be associated with better performance (Familiarity Hypothesis).

## Methods

### Design and procedure

Before the experimental trials, participants were asked to report their age, sex, and level of education. They were also asked how many foreign countries they had traveled to, taken as a proxy for familiarity with laughter from other cultures. Participants were not asked to list the specific countries they had visited as it was assumed that participants would most likely have traveled primarily to countries geographically close to the Netherlands (e.g., France, England). Finally, as an exploratory measure, participants were asked how well they expected to perform in the experimental trials. As participants' expectations of their performance were not found to be related to their actual performance, this measure is not discussed further.

The experimental study had a within-participant design with six conditions, reflecting the six nationalities of the laughter stimuli: Dutch, English, French, US American, Japanese, and Namibian. Each stimulus was presented once in a random order that was fixed across participants. On each trial, participants listened to a laugh, and were asked in a six-way forced choice task from which nationality they thought the laughing person came. Participants were free to do the study with headphones or speakers and to set the sound level themselves. The study did not have a time limit. Upon completion of the study, participants were given feedback on how well they had done in the form of a total score of correct answers.

### Stimuli

The study included a total of 24 stimuli, comprising four amused laughs per nationality. The Dutch, English, and Namibian laughter were taken from Sauter (2013); the US American laughter stimuli were taken from Simon-Thomas et al. (2009); the Japanese laughter stimuli were taken from Sauter et al. (in preparation). The French laughter stimuli were recorded in an equivalent way to those of Sauter (2013). All laughs were part of larger sets of recordings of emotional vocalizations. During the recordings, individuals posed laughs, but also laughed spontaneously. Consequently, there was some variability in spontaneity within each set.

The stimuli from each culture were randomly selected from each set of laughs, with the constraints that there is an equal number of male and female tokens of each nationality and that minimally two different speakers were included for each gender for each culture. The stimuli were recorded individually in a soundproof environment and were on average 2.37 (1.16) s long (see Table 1 in the Supplementary Material for average duration per condition).

### Participants

The study was run online on the website of a Dutch popular science magazine (quest.nl) from June 12th to 26th, 2014, and was publicly accessible. Given that the Quest website in general, and the current study in particular, were in Dutch, participants are assumed to have been either Dutch or Belgian (or sufficiently acculturated to regard the Dutch as their in-group).

The study used an opportunistic sample, collecting as many responses as possible in the available time. Participants were asked whether they consented for their anonymous answers to be analyzed for scientific purposes, but were also given the option to participate without allowing scientific analysis of their data. The study was approved by the University of Amsterdam Department of Psychology ethics committee (reference code: 2014-SP-3736). All participants whose data are included in this manuscript provided written informed consent in accordance with the Declaration of Helsinki.

A total of 1,500 participants took part in the online study. Participants were excluded because (a) they did not consent for their test data to be used for scientific purposes (264 participants), (b) errors in the data log (5 participants), (c) they were less than 18 years old (75 participants), or (d) they did not complete the study (342 participants). The remaining 814 participants (527 women, 287 men) had a mean age of 30.87 years (range: 18–75 years).

## Results

### Data processing

**Figure 1:**
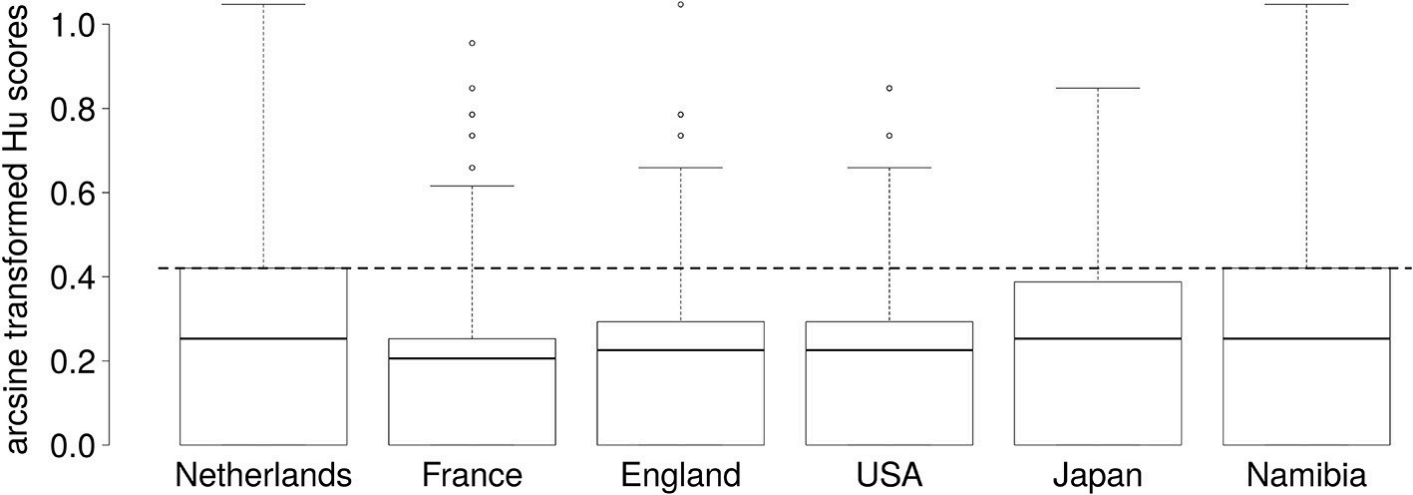
Boxplots of arcsine transformed H_u_ scores for laughter stimuli from each of six countries. The dashed line indicates the chance level. Each box represents the interquartile range, the thick line in each box represents the median score, and the whiskers represent the maximum scores, excluding outliers. No lower whiskers are shown as the minimum scores fall within the interquartile range. Outliers (represented as circles) are scores that were higher or lower than the median by 1.5 times the interquartile range. Outliers were not excluded from any analyses.

To examine performance accuracy, H_u_ scores were calculated (Wagner, 1993). H_u_ scores are unbiased hit rates that correct for response biases, such as disproportionate use of one response alternative. Moreover, H_u_ scores correct for disproportionate presentation of one stimulus type (e.g., presentation of 12 close out-group stimuli vs. 4 in-group stimuli). Raw H_u_ scores range from 0 to 1, with 0 indicating only incorrect classifications, and 1 indicating perfect accuracy. The H_u_ scores for each condition are shown in Figure 1. The H_u_ scores were averaged across all conditions to provide a general measure of performance for each participant. This is referred to as the Mean H_u_ score. For ease of interpretation, the classifications are also provided in Table 1 in percent.

**Table 1:** Confusion matrix of answer proportions in percent.

**Stimulus**	**Netherlands**	**France**	**England**	**USA**	**Japan**	**Namibia**
**JUDGMENT**						
Netherlands	**26.32**	20.76	17.29	13.76	13.73	8.14
France	19.44	**18.52**	14.47	8.51	23.80	15.26
England	18.46	20.64	**18.86**	14.96	8.94	18.15
USA	7.63	17.20	13.45	**17.60**	30.28	13.85
Japan	12.75	21.93	15.14	10.84	**25.80**	13.54
Namibia	15.14	10.29	13.88	27.95	6.70	**26.04**

*Classifications across the diagonal are correct classifications, shown in bold*.

Because H_u_ scores are proportional measures, the scores were arcsine transformed prior to further analysis to stabilize variance and normalize the data (see Wagner, 1993). Following this transformation, all variables were checked for normality with Shapiro–Wilk tests, which indicated that they were not normally distributed (*p*s < 0.001). We therefore employed a non-parametric equivalent of the *t*-test, the Wilcoxon Signed-Rank test for all comparisons between two conditions. For ANOVAs and regression analyses, parametric tests were used, as they are known to be robust against normality violations (Norman, 2010). ANOVAs were employed in all comparisons across three conditions and regressions were used in cases in which the independent variable was not nominal.

In order to allow us to accept or reject the null hypothesis with known certainty, all of the described tests were run with H_u_ scores using both frequentist analyses and the Bayesian equivalents. Frequentist analyses test the probability of the null hypothesis, given the data. Bayesian analyses test the probability of both the alternative and the null hypothesis, given the data. Consequently, conducting Bayesian analyses can yield evidence for either the null or the alternative hypothesis. Bayesian analyses calculate the probability distribution of a parameter (e.g., a difference score) by using the data to update the prior distribution, a parameter distribution based on what is known about the parameter from previous research or theoretical considerations (for an introduction to Bayesian analysis and modeling see Lee and Wagenmakers, 2013). The frequentist analyses were conducted with R (R Core Team, 2013). The Bayesian parametric analyses were run in JASP (The JASP Team, 2017). The non-parametric Bayesian one-sample *t*-tests were run using a computer program by van Doorn et al. (in preparation) The test estimates the effect size δ which is the difference between scores and chance level. The test uses a prior of δ ~ *Cauchy*(0, 1), a t-distribution with a single degree of freedom (Rouder et al., 2009). The Cauchy distribution offers a useful prior because it puts less weight on unrealistic values of δ, and it assumes that small effects occur with greater frequency. Bayes factors were computed with the Savage-Dickey density ratio. If the Bayes factor is greater than 1 then the analysis shows evidence for the alternative hypothesis. If the Bayes factor is lower than 1 then the analysis shows evidence for the null hypothesis. Bayes factors above 100 are considered “extreme evidence for the alternative hypothesis” (Jeffreys, 1961; for more information see Wetzels et al., 2010).

### The specific group identification hypothesis

**Figure 2:**
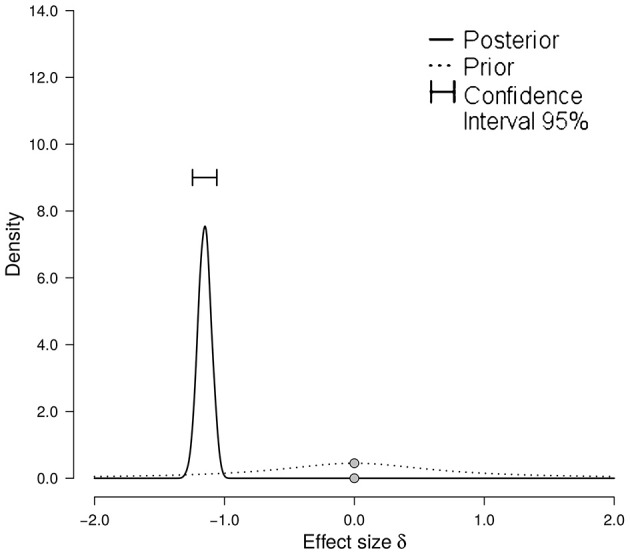
Prior and posterior distribution with Bayesian confidence interval of the effect size δ. The prior distribution (dashed line) shows the distribution expected under the null hypothesis with no data (i.e., performance at chance level). The posterior distribution (solid line) shows the distribution that is expected given the data. The point of interest (zero) is marked with gray dots on both distributions. A score of zero on the x-axis represents performance at chance level.

The Specific Group Identification Hypothesis predicted that participants can accurately infer group membership from laughter, when groups are operationalized as countries. The mean overall H_u_ scores were therefore compared to the chance level (i.e., 1/6). The frequentist test in the form of a Wilcoxon-Signed Rank tests showed that participants performed significantly worse than chance (Median of mean H_u_ score: 0.218, *p* < 0.001, *r* = −0.85). The Bayesian test also showed overwhelming evidence for the alternative hypothesis of participants performing significantly worse than chance. The effect size was estimated to have a median of −1.151 with a Bayesian 95% confidence interval of [−1.246, −1.058]. The prior and posterior distributions can be seen in Figure 2. These tests thus provided no support for the Specific Group Identification Hypothesis.

Although the overall scores clearly showed that performance was below chance levels, participants may have been able to detect laughter from individual countries at better-than-chance levels. Therefore, country-specific H_u_ scores were computed (see Table 2). These were individually compared to chance level using multiple Wilcoxon-Signed Rank tests, Bonferroni corrected for multiple comparisons, and the Bayesian equivalent test. All comparisons showed that the H_u_ scores were significantly below chance and Bayes factors showed that the alternative hypothesis with scores lower than chance was over 1,000 times more likely given the data. These results indicate that participants were not able to accurately infer group identity at the country-level for any of the countries.

**Table 2:** Comparisons of group scores with chance level for Wilcoxon Signed-Rank Test and Bayesian equivalents using arcsine transformed H_u_ scores of laughter from individual countries (above) and grouped countries (below).

**Stimulus origin**	**Median (H_u_ score)**	**Chance level (H_u_ score)**	**Effect size^a^**
Netherlands	0.253	0.421	−0.60
France	0.206	0.421	−0.79
England	0.226	0.421	−0.75
USA	0.226	0.421	−0.77
Japan	0.253	0.421	−0.66
Namibia	0.253	0.421	−0.58
In-Group	0.252	0.421	−0.60
Close Out-Group	0.502	0.784	−0.86
Distant Out-Group	0.361	0.615	−0.85

*All tests were significant at an α-level of 0.001, Bonferroni corrected for multiple comparisons*.

a *Effect sizes are applicable to the frequentist analyses only*.

### Broad group identification hypothesis

Next, we sought to test the Broad Group Identification Hypothesis, which predicted that participants can accurately infer group membership, when operationalized as in-group, close out-group, and distant out-group. H_u_ scores do not control for differing chance levels across conditions. Therefore, in order to test the Broad Group Identification Hypothesis, the difference between H_u_ score and chance level was calculated for each condition. When Dutch laughter was presented, there was only one correct answer out of the six response alternatives, and consequently, the chance level for the in-group was 1/6. For trials in the close out-group condition, there were three correct answers (French, English, US American) out of the six response alternatives. In that condition, the chance level was thus 3/6 (i.e., 1/2). When participants heard laughter from the distant out-group, there were two correct answers (Japanese, Namibian) out of the six response options. Therefore, the chance level was 2/6 (i.e., 1/3). In each condition, chance was subtracted from the H_u_ scores, resulting in difference scores.

A one-way repeated-measures ANOVA was run with the difference scores, comparing performance for in-group (the Netherlands), close out-group (England, France, and USA), and distant out-group (Japan, Namibia). As Mauchly's test indicated violation of the sphericity assumption (*W* = 0.96, *p* = 0.002, η = 0.98), Greenhouse-Geisser corrected scores are reported^1^. Performance differed significantly across the three conditions: *F*_GG(4.9, 3983.7)_ = 43.79, *p* < 0.001. In the Bayesian analyses, the alternative model which allowed differences between conditions was tested against a null model which did not allow for differences. As in the *t*-test, the prior was specified as a Cauchy distribution. There was a significant difference; BF_10_ > 1,000.

**Figure 3:**
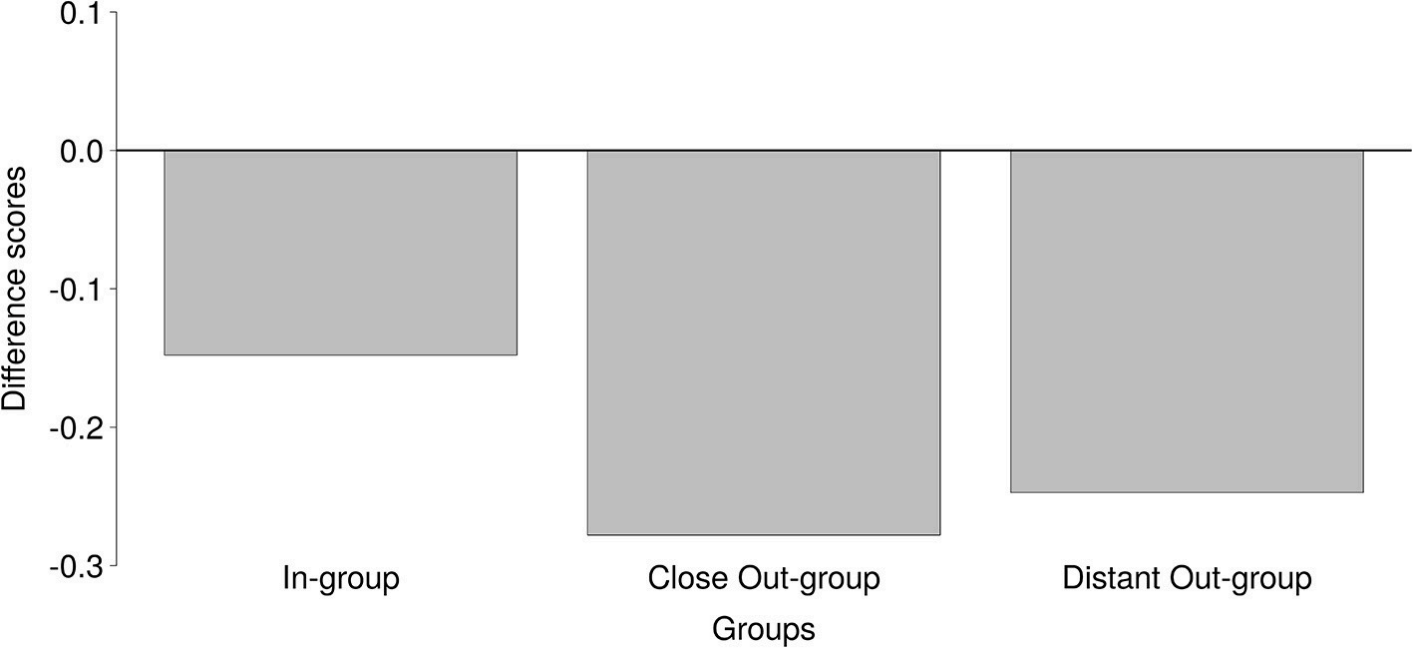
Difference scores (H_u_ scores—chance level) for performance in the separate conditions In-group, Close Out-group, and Distant Out-group. Higher scores represent better performance.

As can be seen in Figure 3, participants performed worse in the close out-group condition compared to the in-group (*V* = 22,482, *p* < 0.001; BF_10_ > 1,000) and distant out-group conditions (*V* = 294,730, *p* < 0.001; BF_10_ > 1,000). Moreover, participants performed better in the in-group condition compared to the distant out-group condition; *V* = 90,108, *p* < 0.001; BF_10_ > 1,000. Yet, in none of the conditions did participants perform better than chance (see Table 2).

### The familiarity hypothesis

We predicted that greater exposure to laughter from members of other cultural groups would be associated with better performance (the Familiarity Hypothesis). There was considerable variability in how many countries participants had visited, with 20.1% having visited 1–5 countries, 39.3% having visited 6–10 countries, 33.5% having been to 11–20 countries, and 7.0% reporting having traveled to 21 or more countries.

A linear model was estimated to check whether the number of countries that participants had visited would predict group identification performance. In the Bayesian analysis, the JASP program uses multivariate generalizations of Cauchy priors on standardized effects with a prior width of 0.5 (see Rouder et al., 2012). The results of both the frequentist and the Bayesian analysis showed that familiarity was not associated with performance [*F*_(3, 810)_ = 1.066, *p* = 0.36; BF_01_ = 7.288]. Note that this Bayes factor denotes the factor in favor of the null hypothesis. The Bayes factor in favor of the alternative hypothesis was BF_10_ = 0.137.

A further exploratory analysis was conducted because we considered it likely that Dutch participants would have mainly traveled to foreign countries that are in the close out-group, such as France or England, compared to countries that are less popular travel destinations from the Netherlands, such as Namibia or Japan. Therefore, we speculated that familiarity may be relevant mainly for the close out-group. We therefore tested whether performance in the close out-group condition was higher for participants with greater exposure to foreign cultures. However, there was no significant association [*F*_(3, 810)_ = 1.93, *p* = 0.12; BF_01_ = 9.430]. The Bayes factor in favor of the alternative hypothesis BF_10_ was 0.11.

## Discussion

This study investigated whether listeners can identify group membership from individual laughter segments. Neither frequentist nor Bayesian analyses yielded any support for participants being able to reliably perform group identification based on laughter sounds: Participants consistently performed below chance levels. Participants performed especially poorly with close out-group laughs (from England, France, and USA), compared to in-group laughs (from the Netherlands) and distant out-group laughs (from Japan and Namibia), but in no case did performance exceed chance. The current study also asked whether variability in participants' exposure to other cultures would be linked to their performance. However, neither frequentist nor Bayesian analyses yielded support for this prediction either: no association was found between familiarity and group identification performance. It is worth acknowledging, however, that our measure of familiarity was indirect (number of foreign countries visited) and thus did not directly probe whether participants had visited the countries included in the current study.

These results support the findings of Sauter (2013), which showed that listeners were unable to judge group membership from non-verbal vocalizations, including laughter. However, previous research has found that perceivers can accurately judge group membership from facial expressions (Marsh et al., 2003) and language dialects (e.g., Kerswill and Williams, 2002). It is worth noting that task complexity may have played a role. In the study by Marsh and colleagues, in which participants differentiated Japanese-American and Japanese faces, participants performed a two-way forced choice (Marsh et al., 2003). In the current study, participants performed a six-way forced choice. The current set of results does not rule out the possibility that the accents in emotional expressions are sufficient to communicate whether a signal is from one's own, as opposed to another, group, but little beyond that.

Another possibility is that facial, but not vocal cues, provide group identity cues. This seems unlikely, given that spoken language is strongly connected to social identity (Giles and Viladot, 1994), and accents differ sufficiently between groups for others to use it for accurate group classification (Kerswill and Williams, 2002). Observers even preferentially rely on a speaker's linguistic dialect compared to their visual appearance (Rakić et al., 2011). However, this clear encoding of identity and group cues may be limited to volitionally produced vocalizations. Volitionally produced vocalizations involve more articulation and more complex coordination than the production of spontaneous laughter (Ruch and Ekman, 2001). A recent study found that speaker identity recognition was impaired for authentic, as compared to volitional, laughter (Lavan et al., 2016), which may reflect differences in vocal production between signals produced under reduced volitional control, such as spontaneous laughter, and volitional vocalizations, such as speech and volitional laughter. Future research could compare spontaneous and posed laughter directly to shed more light on this issue. It may thus be that the cues that listeners use to judge group identity and individual identity are reduced in spontaneous non-verbal emotional vocalizations, including laughter.

The current results point to a potential boundary condition for motivational mechanisms of emotion perception. If perceivers cannot reliably judge group membership from non-verbal emotional vocalizations, this suggests that motivational mechanisms likely do not operate on these kinds of cues. As already shown by Sauter (2013), emotion recognition is superior for in-group non-verbal expressions. This indicates that vocalizations from different groups are not identical, and that these dialects in expressions are sufficient for the in-group advantage to occur in the absence of motivational factors. This does not mean that motivational mechanisms do not operate in cases where a perceiver is able to infer the group membership of the expresser, such as for example, for facial expressions.

## Author contributions

MR analyzed the data. DS designed the study and supervised the analysis. Both authors interpreted the results, wrote the manuscript, and approved prior to submission. The authors agree to be accountable for the content of this work.

### Conflict of interest statement

The authors declare that the research was conducted in the absence of any commercial or financial relationships that could be construed as a potential conflict of interest.

## Footnotes

^1^As Greenhouse-Geisser corrected ANOVAs can suffer from lower power, the analysis was rerun using a multilevel approach that is not affected by sphericity violations. The pattern of results was identical to those reported in the main text.
