# Supplementary material for: Telling Friend from Foe: Listeners Are Unable to Identify In-Group and Out-Group Members from Heard Laughter
Source: Front Psychol. 2017 Nov 16;8:2006. doi: 10.3389/fpsyg.2017.02006 (PMC5696792; doi:10.3389/fpsyg.2017.02006)
Supplement: Supplementary file 1 [file Table1.DOCX]

Supplementary Table 1.

Average duration of the laughter segments for each country.

| Duration (s) | |
| --- | --- |
| England | 1.82 (0.39) |
| Netherlands | 2.62 (1.28) |
| France | 1.12 (0.49) |
| Japan | 3.36 (1.89) |
| Namibia | 2.13 (0.97) |
| USA | 3.14 (1.92) |
| Average | 2.37 (1.16) |
